# Combining physicochemical properties and microbiome data to evaluate the water quality of South African drinking water production plants

**DOI:** 10.1371/journal.pone.0237335

**Published:** 2020-08-13

**Authors:** Tawanda E. Maguvu, Cornelius C. Bezuidenhout, Rinaldo Kritzinger, Karabo Tsholo, Moitshepi Plaatjie, Lesego G. Molale-Tom, Charlotte M. Mienie, Roelof D. Coertze

**Affiliations:** Unit for Environmental Sciences and Management - Microbiology, North-West University, Potchefstroom, South Africa; Cairo University, EGYPT

## Abstract

Anthropogenic activities in catchments used for drinking water production largely contaminates source waters, and this may impact the quality of the final drinking water product. These contaminants may also affect taxonomic and functional profiles of the bacterial communities in the drinking water. Here, we report an integrated insight into the microbiome and water quality of four water treatment plants (NWC, NWE, WCA and NWG) that supply portable water to communities in South Africa. A new scoring system based on combined significant changes of physicochemical parameters and microbial abundance from raw to treated water was used to evaluate the effectiveness of the treatment plants at water purification. Physicochemical parameters which include total soluble solids, turbidity, pH, nitrites and phosphorus among others, were measured in source, treated, and distributed water. There were general statistically significant (P ≤ 0.05) differences between raw and treated water, demonstrating the effectiveness of the purification process. Illumina sequencing of the 16S rRNA gene was used for taxonomic profiling of the microbial communities and this data was used to infer functional attributes of the communities. Structure and composition of the bacterial communities differed significantly (P < 0.05) among the treatment plants, only NWE and NWG showed no significant differences (P > 0.05), this correlated with the predicted functional profile of the microbial communities obtained from Phylogenetic Investigation of Communities by Reconstruction of Observed States (PICRUSt), as well as the likely pollutants of source water. *Bacteroidetes*, *Chlorobi* and *Fibrobacteres* significantly differed (P < 0.05) between raw and distributed water. PICRUSt inferred a number of pathways involved in the degradation of xenobiotics such as Dichlorodiphenyltrichloroethane, atrazine and polycyclic aromatic hydrocarbons. More worryingly, was the presence of pathways involved in beta-lactam resistance, potential pathogenic *Escherichia coli* infection, *Vibrio cholerae* infection, and Shigellosis. Also present in drinking and treated water were OTUs associated with a number of opportunistic pathogens.

## Introduction

Water quality in several parts of South Africa is threatened by urbanization (poorly or untreated sewage and polluted storm water), mines (effluents containing metals and acid), agriculture (return flow that contain excessive amounts of pesticides, herbicides and fertilizers) and various industries [1]. This is particularly the case for the Vaal River system that became the receptacle of pollutants through runoff and infiltration [2]. These anthropogenic activities results in poor quality of most source waters which in turn will require sophisticated systems and additional purification steps for the delivery of good potable drinking water [3]. These sophisticated systems are costly to operate and they are not always available [4]. Poor management and maintenance of existing drinking water infrastructures may also lead to the degradation of drinking water quality even if the raw water is of reasonable quality [5]. To encourage municipalities and water utilities in South Africa to manage and maintain infrastructure, the Drinking Water Quality Framework for South Africa was introduced [6]. This is an incentive-based water regulation and monitoring framework that is defined by the Blue Drop Certification Programme for Drinking Water Quality Management Regulation [1]. The regulation and monitoring of drinking water quality is based on legislated norms and standards such as the South African National Standards [6–8].

The physical and chemical properties of water intended for drinking and other domestic purposes must not exceed specified limits [9]. These physical and chemical properties may affect the appearance, colour and odour of the water to levels which are unacceptable to the consumers regardless of not posing any dangers. Consumers have the right to evaluate the quality and acceptability of the water [10]. The potential for drinking water to transport and disseminate microbiological pathogens to consumers, is a reality, for example the 2015 cholera outbreak caused by drinking contaminated water in Kasase, Uganda [11]. For this reason, physical and chemical processes are used to remove these pathogens from drinking water, and to evaluate the efficacy of the processes, surrogate organisms such as the bacterium from faecal origin, *Escherichia coli* is used [1, 10, 12]. Water intended for human use and consumption must be free of any faecal or *E*. *coli* indicator organisms [13]. Drinking water distribution systems are, however, not isolated, sterile environments and may contain heterotrophic bacteria. These include all bacteria that use organic nutrients for growth and are universally present in all types of water systems. Heterotrophic plate count bacteria, a subset of heterotrophic bacteria are thus found in drinking water and up to 10^3^ cfu/ml is allowed in a small number of samples [14–16].

Next generation sequencing (NGS) of the 16S rRNA gene in microbial community environmental DNA demonstrated that safe, high-quality drinking water contains a unique biodiversity [17, 18]. These communities are impacted by the quality of the source water, purification process, materials used in the distribution system, and physical forces in the system [17, 18]. Elevated water temperatures, low residual chlorine and nutrients (carbon, phosphorus, nitrogen, and iron) are important factors for maintaining microbial communities in drinking water distribution systems [17]. The 16S rRNA gene profile data are informative at the population and community level. It can be processed into various ecological diversity indices [17, 18]. In addition, phylogenetic datasets generated through NGS of the 16S rRNA can be used for extrapolating metabolic and ecosystem functions [19].

The aim of the present study was thus to provide insights into the microbiome and water quality of selected drinking water production plants in South Africa and to discuss the potential application of such data in interpreting the impact of anthropogenic activities on the process and cost of water purification. At the same time we were evaluating the efficacy of the treatment process used by the drinking water treatment plants. More importantly, we introduce a new method which allows for combined evaluation of physicochemical parameters and microbiome data to evaluate the efficacy of different drinking water treatment plants to remove contaminants.

## Materials and methods

### Water treatment plants, and sample collection

Table 1 shows a summary of information of the various drinking water treatment plants (DWTP) used in this study. Samples were collected in June 2017 from raw, treated and distributed water of each DWTP following the Department of Water Affairs and Forestry (DWAF) sampling guidelines [20]. Sampling was done in triplicates, briefly, the samples were collected in sterile 1 litre Schott bottles, and they were stored and transported on ice. All samples were subjected to laboratory analyses within 8 hrs of sample collection. Table 1 also shows the source of raw water, anthropogenic activities likely to have an influence on the quality of raw/source water of all the treatment plants. The exact locations and names of the treatment plants were anonymised as the results might influence the consumer’s opinions. The four treatment plants were designated as WCA, NWC, NWE and NWG. For sampling WCA, NWC and NWE treatment plants, written permissions were obtained from the local municipalities, and the municipalities serves as both water service providers and water service authority. For sampling NWG treatment plant, written permissions were obtained from both the water service provider (a private company) and the water service authority (local municipality). For distributed samples, non-written permission for sampling was obtained from the household where the samples were collected. In South Africa, the water service authorities are under the jurisdiction of the Department of Water Affairs and Sanitation formerly DWAF.

**Table 1:** The different drinking water treatment plants and their treatment processes.

	Water Sources	Purification/Treatment processes	Plant Capacity (Mℓ/day)	Population served	Land use issues
**WCA**	Surface (Dam), Ground (Boreholes), WWTP Effluent	Surface and ground–Sand filtration–Chlorination	4.92	51 080	Agriculture
		WWTP effluent–Sedimentation—Sand filtration–UF–RO—Advanced Oxidation–Chlorination	2.5		
**NWC**	Eye (Natural spring)	Sand filtration- Chlorination	14	56 702	Agriculture, uncovered canal
**NWE**	Surface (Dam), Ground (Borehole)	Coag/Flocc—Sedimentation- Sand filtration, Activated carbon filtration—Chlorination	33.6	162 762	Agriculture, Informal settlements Urbanization, Mining, uncovered canal
**NWG**	Surface (River)	Coag/Flocc–Dissolved Air Floatation–Ozonation—Sedimentation- Sand filtration, Chlorination	250	417 282	Agriculture, Informal settlements Urbanization, Mining,

Coag/Floc–coagulation and flocculation; WWTP–waste water treatment plant; RO–Reverse osmosis; UF–ultra filtration

### Analysis of physicochemical parameters

Water quality parameters (pH, temperature and total dissolved solids) were measured *in situ* using a multi- 350 probe analyser (Merck, Germany). Turbidity was measured using a HACH 21000P Turbidity meter (HACH, USA). A HACH DR 2800 spectrophotometer (HACH, USA) was used to measure phosphates, nitrate, nitrite and free chlorine. Microsoft Excel (2016; version 16.0.6868.2067) was used to determine the averages and standard deviations. Correlations were made between the physicochemical parameters of raw, treated and distributed water by Principal Component Analysis (PCA) using Canoco software version 4.5.

### 16S rRNA gene profiling

Environmental DNA was extracted using the Power Water DNA isolation kit (MoBio, US) following the manufacturer’s protocol. DNA concentration was quantified using a Nanodrop 1000 spectrophotometer (Thermo Fisher Scientific, USA). Amplification of the V3 and V4 variable region of the 16S rRNA gene was done using universal primers 341F and 805R [21]. PCR reactions for each sample were performed in duplicate and contained 1 μL of normalised DNA (20 ng/ μL), 0.2 μM of each forward and reverse primer, 1.25 U HotStar HiFidelity Polymerase, HotStar HiFi buffer (Qiagen, Germany), and nuclease free water to give a total volume of 25 μL. The following thermal cycling conditions were used; initial denaturing 95°C for 5 mins, followed by 35 cycles of amplification at 95°C for 30s, annealing 55°C for 60s, extension 72°C for 60 seconds, and final extension 72°C for 10 mins. Amplicons were purified using Agencourt AMPure XP beads (Beckman Coulter, USA) following the manufacturer’s protocol. Nextera XT indexing primers (N7xx and S5xx) were attached in a subsequent PCR reaction using 2xKAPA HiFi HotStart Ready Mix and thermal cyling conditions of 95°C for 3 minutes followed by 8 cycles of 95°C for 30 seconds, 55°C for 30 seconds, 72°C for 30 seconds and a final elongation step of 72°C for 5 minutes. The success of the PCR was determined by agarose gel electrophoresis. Index PCR products were subjected to a second clean-up step as described above. The libraries were then quantified using a Qubit fluorometer (Qubit 3.0, Life Technologies, Malaysia) and normalised to 4 nM. The final library was generated by pooling 5 μl of each sample, as well as a 10% spike-in with a PhiX control library. The libraries were analysed using a MiSeq reagent kit v3 600 cycles. A 2x 300 bp paired-end sequencing was performed on a MiSeq Illumina platform (Illumina Inc. CA, USA) at the North-West University sequencing facility.

### Bioinformatics analysis and data visualisation

Overlapping paired-end Illumina fastq files were merged using the PANDAseq assembler [22], and reads were quality checked using FastQC (Babraham Bioinformatics, UK; https://www.bioinformatics.babraham.ac.uk/), where necessary trimming was done using ea-utils. Downstream analysis was done using Quantitative Insights into Microbial Ecology (QIIME 1.91) [23]. Merged quality-filtered reads were clustered into operational taxonomic units (OTUs) at 97% 16S rRNA gene similarity using UCLUST algorithm [24] against the Greengenes database. The version gg_13_5 was used for closed reference OTU picking which were used for analysis with Phylogenetic Investigation of Communities by Reconstruction of Unobserved States (PICRUst), while the version gg_13_8 was used for open reference OTU picking. Closed reference OTUs were only used for PICRUst analysis. The taxonomy of each phylotype was classified based on the Greengenes database using the Ribosomal Database Project. For visualisation and statistical analysis, the OTUs were subjected to Microbiome Analyst [25], METAGENassist [26], PICRUSt [27] and Statistical Analysis of Metagenomic Profiles (STAMP) [28].

### Comparative statistical analysis of selected physicochemical properties and microbiome data

The reduction of total dissolved solids (TDS), turbidity, phosphates, nitrites, nitrates and OTUs from raw water to final drinking water for each water purification plant was evaluated. This was achieved by using a scoring system where a score of 0 was assigned if no significant reduction or increase of a parameter was observed; a score of -1 was assigned for a significant increase of a parameter from raw to treated water whereas a score of 1 was assigned for a significant decrease of a parameter from raw to treated water. A total score for each purification plant was calculated in which higher scores indicated better overall functionality of the purification plant. The significant changes between the treated and raw water was calculated using the Student’s *t*-test for each parameter, individually for each purification plant. The *t*-test analysis was conducted using a one-tailed distribution assuming unequal variances, and statistical significance was recognized for P-values < 0.05.

## Results

### Physicochemical analysis

Turbidity in the raw water (0.59 ± 0.6 NTU) of the NWC treatment plant was significantly lower (P < 0.05) than in the treated water (1.21 ± 0.8 NTU) as well as that of water in the distribution system (1.57 ± 1.06 NTU) (Table 2). In contrast, turbidity significantly (P ≤ 0.05) decreased from raw to treated water at the NWE and NWG treatment plants. At the WCA treatment plant, there was no significant difference in turbidity between raw and treated water (P > 0.05) however, treatment resulted in a decrease of turbidity (Table 2). In the distributed water, at NWC and NWG treatment plants turbidity (1.57 ± 1.06 and 1.02 ± 1.91 NTU, respectively) was higher when compared to that in the distributed water at WCA and NWE (0.51 ± 0.08 and 0.46 ± 0.19 NTU), respectively (Table 2).

**Table 2:** The physical parameter measurements of the DWTPs.

		Turbidity (NTU)			pH			Temperature (°C)			TDS (mg/L)		
Sampling site		Raw	AT	Dis	Raw	AT	Dis	Raw	AT	Dis	Raw	AT	Dis
**WCA**	Minimum	0.1	0.1	0.4	7.1	6.4	7.7	11.8	12.3	15.6	217	143	713
	**Average**	**3.27±32.48**	**0.36±0.36**	**0.51±0.08**	**7.81±0.32**	**7.49±0.1**	**7.90±0.15**	**16.0±2.63**	**15.94±3.35**	**15.83±0.21**	**732± 327.02**	**664±322.47**	**833±90.87**
	Maximum	9.7	0.6	0.7	8.3	8.0	8.2	19.1	21.0	16.0	989	935	915
**NWC**	Minimum	0.2	0.2	0.4	8.6	8.5	8.5	15.7	15.4	13.9	332	333	317
	**Average**	**0.59±0.60**	**1.21±0.80**	**1.57±1.06**	**8.67±0.10**	**8.60±0.08**	**8.62±0.09**	**17.8±2.20**	**16.9±1.45**	**20.2±3.34**	**363±23.20**	**365±23.81**	**362±23.16**
	Maximum	1.8	2.4	3.8	8.8	8.7	8.8	21.2	18.8	26.7	382	387	382
**NWE**	Minimum	0.2	0.3	0.0	7.8	7.4	7.4	11.8	12.5	13.0	434	444	393
	**Average**	**2.56±1.20**	**1.21±0.73**	**0.46±0.19**	**8.33±0.36**	**8.15±0.36**	**8.28±0.38**	**17.6±3.80**	**18.6±3.75**	**17.9±2.85**	**493±38.65**	**503±38.23**	**490±45.64**
	Maximum	4.1	2.1	0.7	8.9	8.6	9.2	22.5	24.0	24.6	543	553	547
**NWG**	Minimum	11.4	0.3	0.2	8.4	8.2	8.0	12.2	11.3	14.7	447	453	416
	**Average**	**16.16±3.01**	**1.15±1.42**	**1.02±1.91**	**9.29±0.38**	**8.43±0.23**	**8.47±0.35**	**17.5±4.96**	**18.6±5.37**	**19.8±4.3**	**494±52.75**	**508±58.22**	**533±45.45**
	Maximum	20.6	3.9	8.1	9.7	8.7	9.2	23.3	24.4	26.5	566	586	586

AT–after treatment; Dis–distribution; TDS–total dissolved solids

There were no significant differences in total dissolved solids (TDS) between raw and treated water at all the treatment plants. The WCA treatment plant had a relatively higher TDS (above 600 mg/L) in all the compartments in comparison to the other treatment plants where TDS concentrations were below 600 mg/L (Table 2). The maximum concentration of TDS recorded at the WCA plant was above 900 mg/L (Table 2). Phosphate levels between raw and treated water were not significantly different at three of the four treatment plants. The only exception was the NWG treatment plant where the levels of phosphates were significantly lower (P < 0.05) in the treated water compared to the raw water (Tables 3 and 4). Overall, the WCA treatment plant had higher concentration of phosphorus in all compartments (Table 3).

**Table 3:** The chemical parameter measurements of the DWTPs.

		Phosphorus (mg/ℓ)			Nitrate (mg/L)			Nitrite (mg/ L)			Free chlorine (mg/ L)
Sampling site		Raw	AT	Dis	Raw	AT	Dis	Raw	AT	Dis	Dis
**WCA**	Minimum	1.8	1.0	1.8	0.0	0.8	0.6	0.0	0.0	0.0	0.0
	**Average**	**4.08±1.11**	**3.66±0.11**	**3.67±0.93**	**2.99±3.36**	**1.43±0.63**	**1.11±0.32**	**0.12±0.21**	**0.05±0.08**	**0.14±0.25**	**0.04±0.03**
	Maximum	5.8	5.3	5.0	9.6	2.8	1.6	0.6	0.2	0.8	0.1
**NWC**	Minimum	0.0	0.1	0.1	1.0	0.3	0.8	1.0	0.0	0.0	0.1
	**Average**	**0.79±1.15**	**1.15±1.47**	**0.51±0.62**	**2.33±1.05**	**2.08±0.82**	**2.05±0.66**	**3.58±1.38**	**2.75±2.01**	**2.97±2.06**	**0.37±0.22**
	Maximum	3.5	4.5	2.5	4.7	3.4	3.6	5.0	6.0	11.0	0.7
**NWE**	Minimum	0.3	0.0	0.3	0.0	0.0	0.0	0.0	0.0	0.0	0.0
	**Average**	**1.93±1.92**	**1.79±2.09**	**1.66±0.93**	**0.97±1.31**	**0.71±0.97**	**0.39±0.54**	**0.51±0.75**	**1.00±1.89**	**0.79±1.28**	**0.20±0.39**
	Maximum	5.5	5.1	4.3	4.3	2.9	1.7	2.0	5.0	5.0	1.8
**NWG**	Minimum	0.3	0.2	0.0	0.0	1.3	0.7	1.0	3.0	1.0	0.0
	**Average**	**3.51±2.97**	**1.59±1.50**	**1.12±1.31**	**0.53±0.56**	**2.83±1.17**	**1.93±0.71**	**3.20±3.65**	**5.75±2.73**	**3.75 ±1.44**	**0.21±0.36**
	Maximum	10.2	4.9	4.8	1.4	4.5	3.2	11.0	13.0	8.0	1.2

AT–after treatment; Dis–distribution; COD–chemical oxygen demand

**Table 4:** Evaluation of water treatment effectiveness among the various DWTPs.

		TDS	Turbidity	Phosphates	Nitrites	Nitrates	OTUs	Total Score
**WCA**	P-values	0.266	0.061	0.203	0.136	0.135	0.011*	
	PAT (%)	113.54	14.02	84.93	100.00	39.52	50.81	1
	Score	0	0	0	0	0	1	
**NWC**	P-values	0.431	0.002*	0.366	0.159	0.331	0.179	
	PAT (%)	100.79	210.95	125.93	70.00	90.12	82.42	-1
	Score	0	-1	0	0	0	0	
**NWE**	P-values	0.428	0.004*	0.466	0.476	0.029*	0.024*	
	PAT (%)	100.65	20.50	104.62	92.22	47.62	74.38	3
	Score	0	1	0	0	1	1	
**NWG**	P-values	0.399	0.000*	0.029*	0.005*	0.000*	0.008*	
	PAT (%)	101.75	3.38	42.92	525.00	679.31	38.26	1
	Score	0	1	1	-1	-1	1	

* Indicates significance (P < 0.05); PAT—Percentage after Treatment; TDS–total dissolved solids

The concentration of nitrates in treated water at the WCA and NWC treatment plants did not significantly differ with the concentrations recorded in source water. However, at NWE the treatment of source water resulted in a significant decrease (P < 0.05) of nitrates. In contrast at the NWG treatment plant, the treated water had a significantly (P < 0.05) higher concentration of nitrates than raw water (Tables 3 and 4). There were no significant differences in the concentration of nitrites between raw and treated water at WCA, NWE and NWC treatment plants. On the other hand, at the NWG treatment plant there was a significantly (P < 0.05) higher concentration of nitrites in the treated water compared to the raw water (Table 4). Raw, treated and distributed water was alkaline at all the treatment plants (Table 2). There were no significant differences among the treatment plants as well as within the different compartments of the treatment plants. Free chlorine concentrations in the distributed water were generally low. However, at NWE and NWG, maximum concentrations exceeded 1 mg/L.

**Fig 1:**
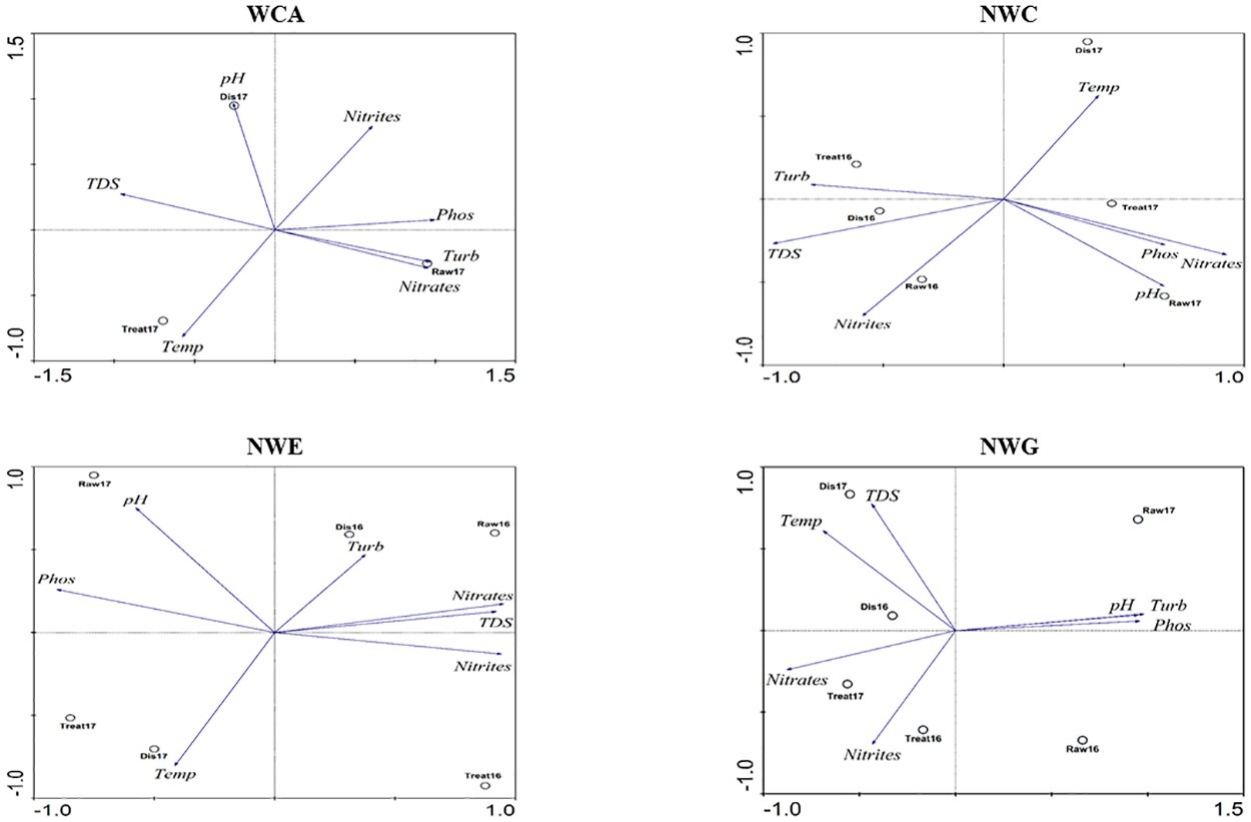
PCA biplots showing the correlation between the various physicochemical parameters with raw, treated and distributed water at all the DWTPs. Raw represents raw water, treat represents treated water and Dis represents distributed water. TDS is total dissolved solids, Turb is turbidity, Temp is temperature and Phos is Phosphates.

Principle component analysis (PCA) of physicochemical properties showed that, at the WCA treatment plant, turbidity and nitrates strongly correlated with raw water, whereas the drinking water was associated with temperature and pH (Fig 1). At NWC raw water correlated with nitrites and pH. Temperature, turbidity and TDS strongly correlated with drinking water (Fig 1). Raw water at NWE correlated mostly with pH, whereas drinking water mostly correlated with turbidity and temperature. Raw water at NWG did not have a specific positive correlation with any parameter whereas the drinking water correlated with nitrates, nitrites, temperature and TDS (Fig 1).

### Taxonomic profiles

**Fig 2:**
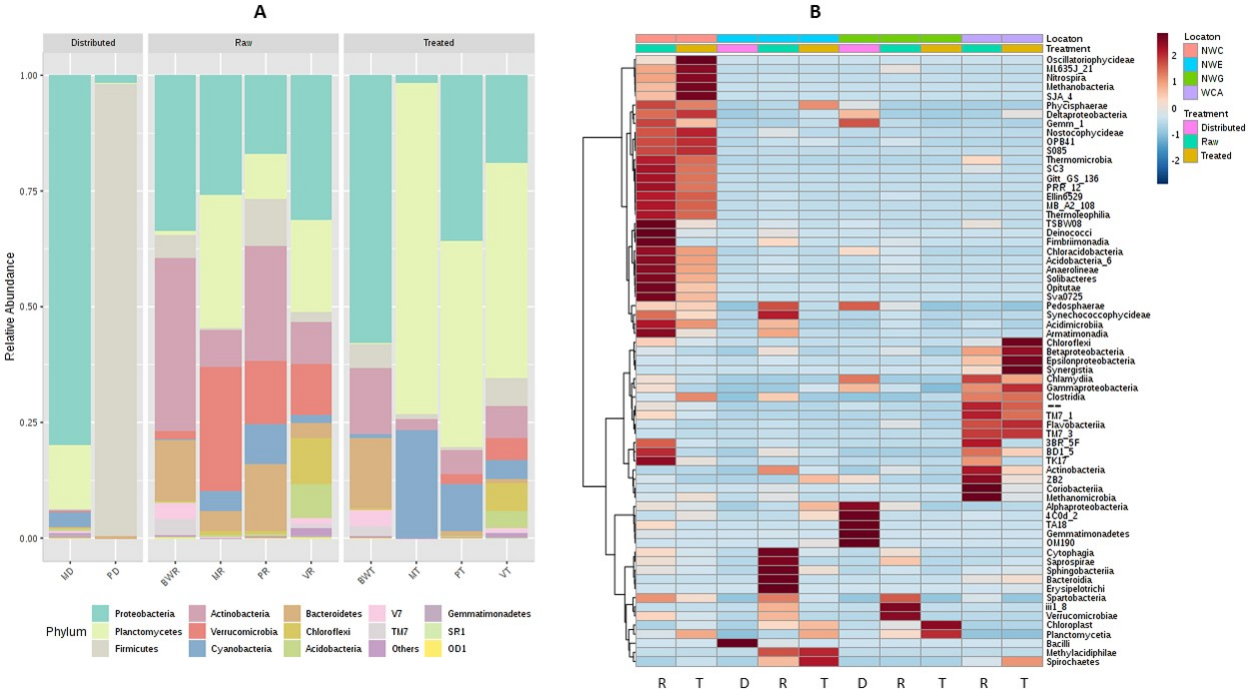
**(A).** A stacked bar plot showing relative abundance of bacterial phyla in source, treated and distributed water of the DWTPs. Only phyla which had an abundance of > 2% are shown. **(B).** A heat map showing the abundance of different bacterial order in source, treated, and distributed water of the DWTPs. R represents raw water, T represents treated water, and D represent distributed water.

Taxonomic classification of the clustered OTUs revealed the presence of 34 bacterial phyla with > 2% abundance. The following trends were observed: At the WCA plant raw and drinking water was dominated with bacteria from the phyla: *Proteobacteria* (raw = 36.63%; treated water = 60.30%), *Actinobacteria* (raw = 35.76%; treated water = 13.64%), *Bacteroidetes* (raw = 13.02%; treated water = 13.64%), 13.02%), *Firmucutes* (raw = 5.68%; treated water = 5.16%) and *TM7* (raw = 3.51%; treated water = 2.13%) (Fig 2). From raw to treated water, *Proteobacteria* increased and *Actinobacteria* decreased whereas the OTUs for other phyla were not substantially affected. At the NWC treatment plant, raw water was dominated with bacteria from the phyla *Proteobacteria* (raw = 31.51%; treated water = 18.61%), *Actinobacteria* (raw = 7.79%; treated water = 6.29%), *Bacteriodetes* (raw = 4.51%; treated water < 2.0%), *Firmicutes* (raw = 2.19%; treated water = 6.60%). In contrast to WCA, the phyla *Planctomycetes Verrucomicrobia*, *Chloroflexi*, *Acidobacteria* and *Cyanobacteria* had > 2.0% abundance (Fig 2). Moreover, *Proteobacteria* OTU levels in the treated water was higher than in raw water. At NWE the following trend was observed: *Proteobacteria* (raw = 18.01%; treated water = 36.80%), *Actinobacteria* (raw = 20.53%%; treated water = 4.97%), *Firmicutes* (raw = 15.82% %; treated water = < 2%) and *Bacteroidetes* (raw = 14.64%; treated < 2%). OTUs for the phyla *Verrucomicrobia*, *Planctomycetes* and *Cyanobacteria* were also greater than 2%. Raw water at the NWG plant was dominated by bacteria of the phyla *Proteobacteria* (27.11%), *Actinobacteria* (7.63%), *Bacteroidetes* (5.50%) *Planctomycetes* (26.84%), *Verrucomicrobia* (25.59%), and *Cyanobacteria* (4.17%). Treated water consisted of *Actinobacteria* (2.55%) *Planctomycetes* (69.44%) and *Cyanobacteria* (23.82%) (Fig 2).

**Fig 3:**
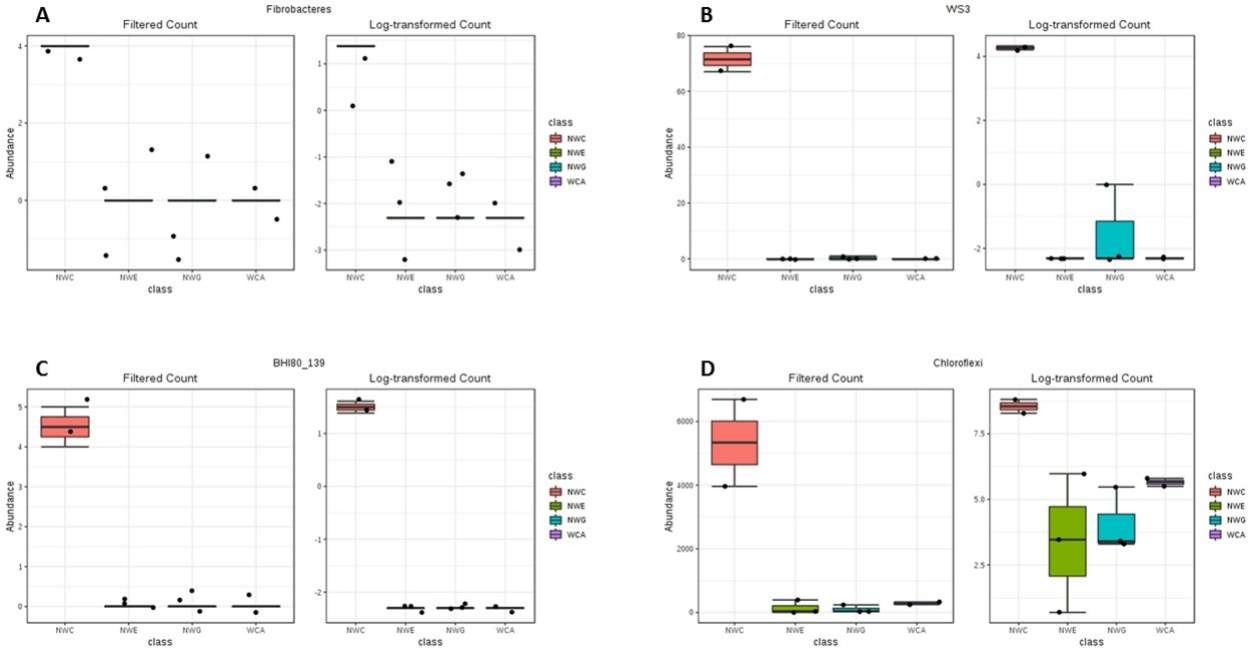
Statistically significant differences (P < 0.05) in bacterial phyla among the treatment plants. A—I Phyla which was significantly higher at the NWC treatment plant compared to all the other plants. J—L Phyla significantly higher at the WCA treatment plant.

Statistical analysis of the 16S profiles showed that phyla WS2, WS3, WS5, Acidobacteria, BHI80-139, BRC1, Chloroflexi, Fibrobacteres, GNO4, Nitrospirae, NKB19, TM6 at the NWC plant were significantly (P ≤ 0.05) higher than at the other treatment plants (Fig 3). The WCA treatment plant had a significantly (P ≤ 0.05) higher proportion of SR1, TM7 and Euryarchaeota (Fig 3). Across plants raw water had a significantly (P = 0.032) higher proportion of Chlorobi. In addition, raw water had significant (P < 0.05) proportions of Fibrobacteres, Bacteroidetes in comparison with distributed water, but no significant differences when compared to treated water.

Treated and distributed water samples were screened for potentially pathogenic bacteria. A number of pathogenic signatures which includes the genera *Acinetobacter*, *Clostridium*, *Legionella*, *Pseudomonas* and *Serratia Tatlockia* were identified. The NWC and NWG treated water had all the above mentioned genera. The NWE treated water had at least one OTU belonging to all the genera except for *Serratia*. OTUs in distributed water of NWE were positive for *Acinetobacter* and *Pseudomonas* while distributed water at NWG had *Pseudomonas*, *Tatlockia* and surprisingly a higher number of OTUs belonging to *Legionella* in comparison with treated water. S1 Table shows the distributions of OTUs within various potentially pathogenic bacterial genera. The species from the various genera included: *Acinetobacter* (*A*. spp., *A*. *johnsonii* and *A*. *rhizosphaerae*), *Clostridium* (*Clostridium* spp., *C*. *intestinale*, *C*. *piliforme* and *C*. *bowmanii*), *Legionella* (*L*. spp. and *L*. *pneumophila*), *Pseudomonas* (*P*. spp., *P*. *pseudoalcaligenes* and *P*. *nitroreducens*, *P*. *veronii* and *P*. *fragi)* and *Serratia* (*S*. spp. and *S*. *marcescens*).

### Alpha diversity

Species richness (OTUs 97% similarity) as indicated by Chao1 index was significantly affected by location/DWTP (P < 0.05; Table 5). However, different treatments (raw, treated, and distribution) did not significantly affect species richness (P = 0.36224; Table 6). Additionally, species displayed similar evenness across all treatments as well as sampling location (Shannon Index; P > 0.05) (Tables 7 and 8).

**Table 5:** Chao1 index among locations.

Location	Treatment	Variable	Value	Standard Error
NWC	Raw	Chao1	1732.3	7.6
	Treated	Chao1	1588.9	8.4
NWE	Raw	Chao1	1100.1	16
	Treated	Chao1	833	10.4
	Distributed	Chao1	59	14.7
NWG	Raw	Chao1	567.4	18.1
	Treated	Chao1	394.4	10.2
	Distributed	Chao1	454.8	12.8
WCA	Raw	Chao1	488.3	13.9
	Treated	Chao1	311.3	10.9

P = 0.023771

**Table 6:** Chao1 index among treatments.

Treatment	Location	Variable	Value	Standard Error
Raw	NWC	Chao1	1721.3	7.6
	NWE	Chao1	1105.1	16
	NWG	Chao1	570.4	18.1
	WCA	Chao1	493.3	13.9
Treated	NWC	Chao1	1587.9	8.4
	NWE	Chao1	836	10.4
	NWG	Chao1	394.4	10.2
	WCA	Chao1	314.3	10.9
Distributed	NWE	Chao1	59	14.7
	NWG	Chao1	447.8	12.8

P = 0.36224

**Table 7:** Shannon index among locations.

Location	Treatment	Variable	Value
NWC	Raw	Shannon	6.5
	Treated	Shannon	6.4
NWE	Raw	Shannon	4.6
	Treated	Shannon	3.6
	Distributed	Shannon	0.2
NWG	Raw	Shannon	3.7
	Treated	Shannon	1.4
	Distributed	Shannon	3.7
WCA	Raw	Shannon	3
	Treated	Shannon	3

P = 0.1245

**Table 8:** Shannon index among treatments.

Treatment	Location	Variable	Value
Raw	NWC	Shannon	6.5
	NWE	Shannon	4.6
	NWG	Shannon	3.7
	WCA	Shannon	3
Treated	NWC	Shannon	6.4
	NWE	Shannon	3.6
	NWG	Shannon	1.4
	WCA	Shannon	3
Distributed	NWE	Shannon	0.2
	NWG	Shannon	3.7

P = 0.37957

### OTU diversity and similarity analysis

**Fig 4:**
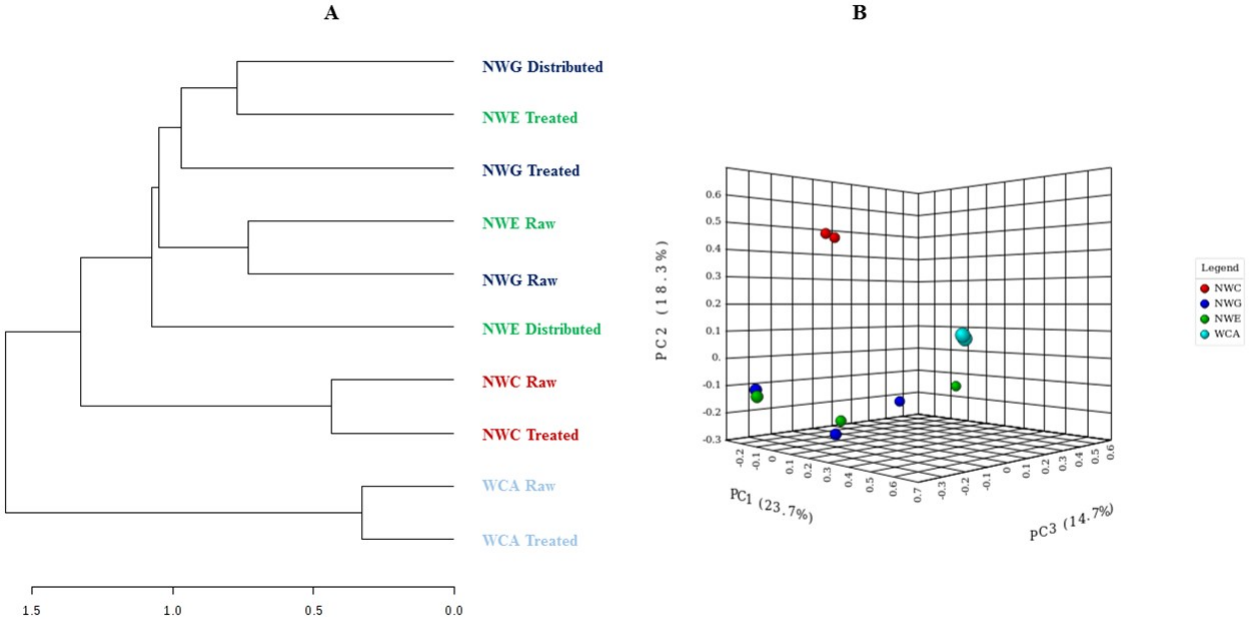
Beta diversity measures. (A) Cluster dendrogram showing how the OTUs from various locations and treatments clusters. Clustering was more by location than treatments. Bray Curtis index measure and ward clustering method was used to generate the dendrogram. (B) PCoA ordination using the Bray Curtis Index and PERMANOVA, distinct clustering which was based on location was observed.

Community OTU comparisons were visualised by PCoA analysis (OTU ≥97% similarity) using Bray Curtis Index (P < 0.05; PERMANOVA; Fig 4). Bray Curtis index showed distinct clustering based on location rather than treatments. A dendrogram generated using the Bray Curtis index distance measure and the Ward clustering algorithm showed that OTUs clustered together mainly by location, rather than treatments. This is consistent with the PCoA plots (Fig 4). WCA raw and treated water formed their own cluster while NWC raw and treated water formed their own sub-cluster. However, NWG and NWE raw water formed their own sub-cluster, with the NWE treated water being slightly distinct. Distributed and treated water at NWG was closely related to NWE treated water (Fig 4).

### Taxonomic-to-phenotype mapping of the OTUs

**Fig 5:**
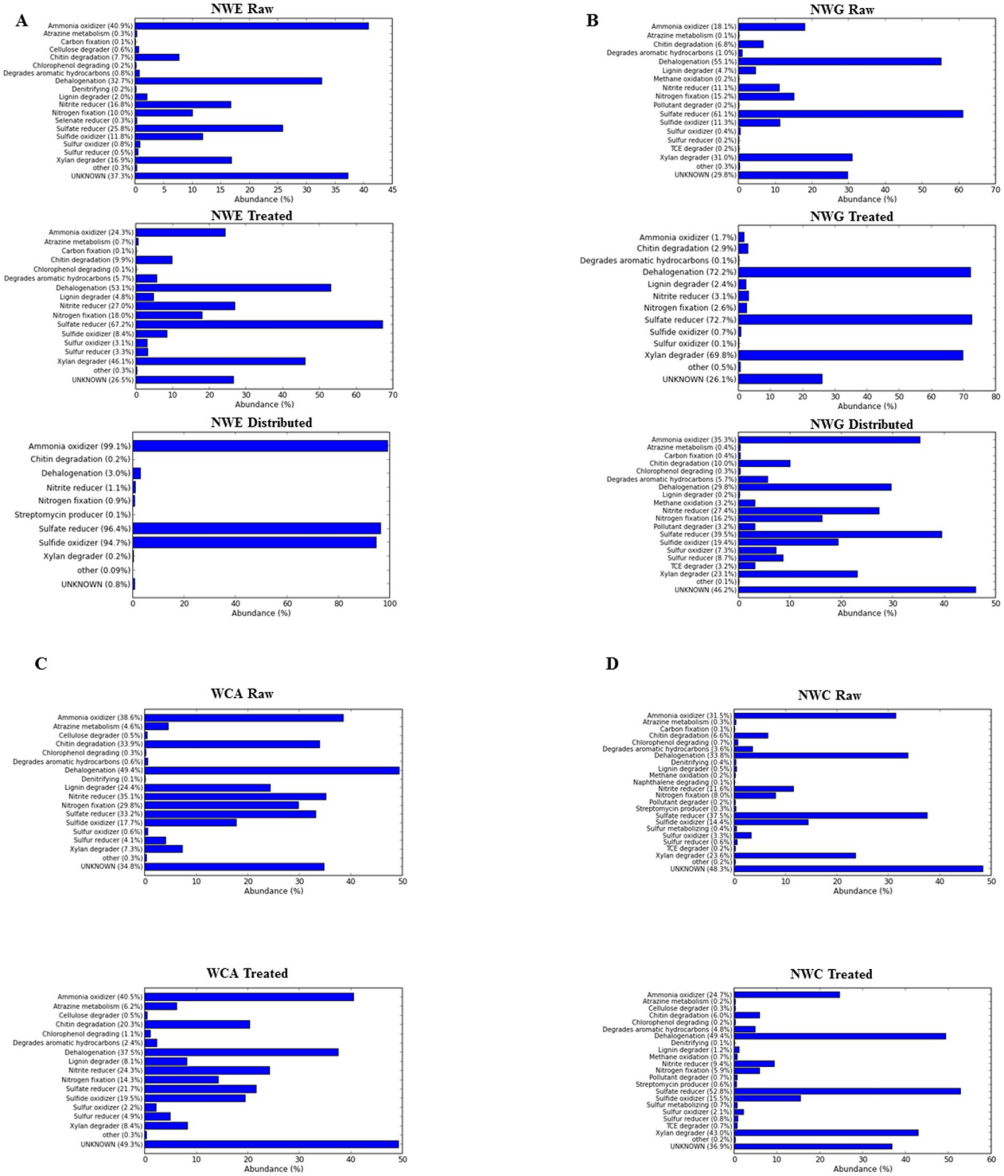
Taxonomic to phenotype mapping of the OTUs using METAGENEasist. Metabolism profiles in raw, treated and distributed water at the NWE treatment plant (A) and NWG treatment plant (B). Metabolism profiles in source and treated water at the WCA treatment plant (C) and NWC treatment plant (D).

Data was normalised by log transformation, and METAGENassist was used for taxonomic-to-phenotype mapping of the OTUs. Abundance of inferred metabolic pathways are shown in (Fig 5). Predicted metabolic pathways for dehalogenation (32.7–55.1%), sulfate reducers (25.8–61.1%) and ammonia oxidizers (18.1–40.9%) were the most dominant pathways in raw water of all plants. Treated water was mainly dominated by predicted metabolic pathways for dehalogenation (37.5–72.2%), sulfate reducers (21.7–72.2%), and xylan degraders (8.4–69.8%). Distributed water was mainly dominated by ammonium oxidizers (35.3–99.1%), sulfate reducers (39.5–96.4%), and sulfite oxidizers (19.4–94.7%). A number of pathways involved in the degradation of xenobiotics which included atrazine degradation, pollutant degrader, degradation of aromatic hydrocarbons and chlorophenol degradation were predicted to be present in all compartments (Fig 5).

### PICRUSt predicted metabolic functions and capacities of the bacterial communities

**Fig 6:**
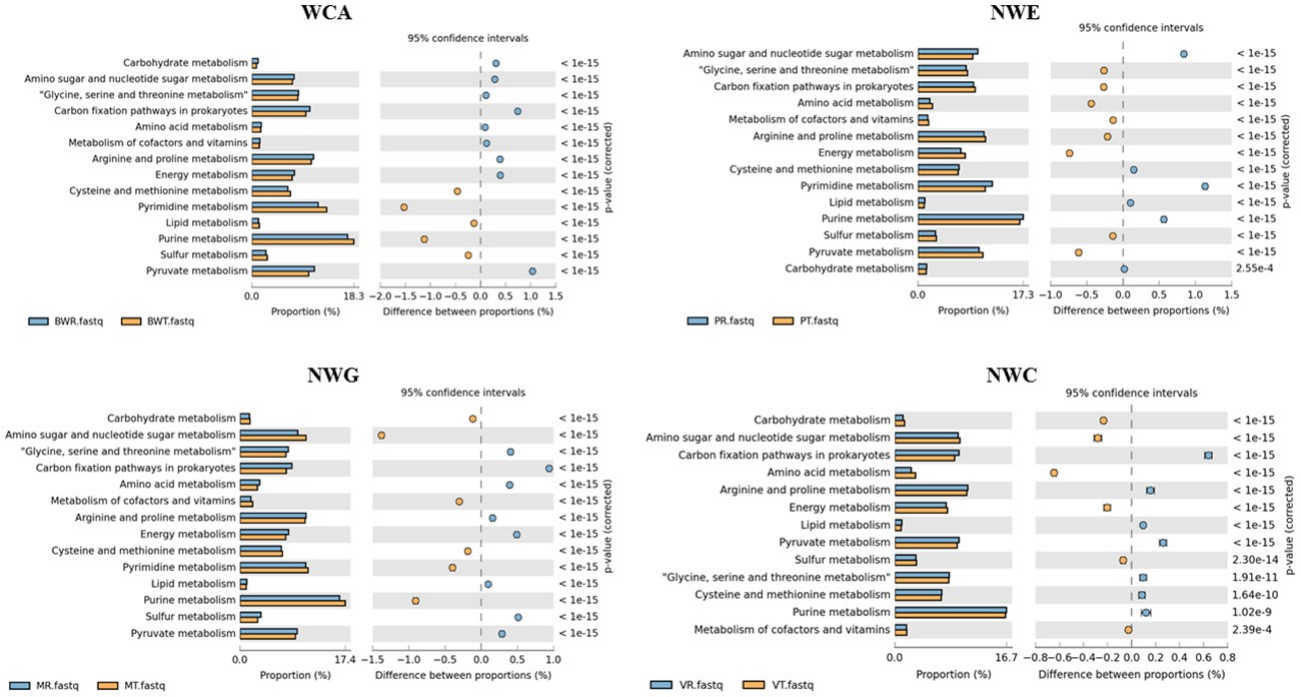
PICRUst predicted metagenomes. Some basic metabolic functions which were statistically significant between raw and treated water of the DWTPs. Blue bars represents raw water while orange bars represents treated water.

PICRUSt prediction of the metabolic functions, was used to have an insight into the role of different microbial communities in source, treated and drinking water from the four different treatment plants. A number of housekeeping pathways which include carbohydrate metabolism, pyruvate metabolism, sulfur metabolism, purine metabolism, lipid metabolism, pyrimidine metabolism, cysteine and methionine metabolism, energy metabolism, arginine and proline metabolism, metabolism of core factors and vitamins, amino acid metabolism, carbon fixation pathways in prokaryotes, glycine, serine and threonine metabolism as well as amino sugar and nucleotide sugar metabolism were predicted and their distribution is shown in (S1 Fig). Fig 6 shows the housekeeping pathways which showed significant differences (P ≤ 0.05) between raw and treated water samples from the same DWTP. Predicted genes which are involved in xenobiotic degradation were also observed. Most notably were those involved in aminobenzoate, polycyclic aromatic hydrocarbons, atrazine, ethylbenzene, fluorobenzoate, naphthalene, dichlorodiphenyltrichloroethane (DDT) degradation. The level of predicted polycyclic aromatic hydrocarbon degradation was significantly higher (P = 0.042) at the WCA treatment plants compared to other locations (S2 Fig). Other predicted xenobiotic degradation pathways did not show any significant difference between or within locations. Pathways involved in beta-lactam resistance were also predicted at all locations and treatments (S2 Fig). Distributed water had a significantly higher (P < 0.05) level of genes predicted to be involved in ABC transporters, arachidonic acid metabolism and transporters. Further important pathways predicted were bacterial chemotaxis, streptomycin biosynthesis, metabolism of xenobiotics by cytochrome P450, pathogenic *E*. *coli* infection, shigellosis (S2 Fig), and *Vibrio cholerae* infection (S2 Fig).

### Comparative statistical analysis of selected physicochemical properties and microbiome data

Evaluation results of the water treatment effectiveness among the various water purification plants are summarized in Table 4. Results indicated no significant reduction or increase of TDS after treatment among the purification facilities. Significant decrease of turbidity was observed for NWG and NWE treatment plants whereas, a significant increase in turbidity was observed at the NWC treatment plant. There were no significant changes in turbidity between raw and treated water at the WCA treatment plant. No significant changes were observed for phosphate removal apart from the NWG plant at which a significant reduction was observed after treatment. There were no significant changes in nitrate removal at all the treatment plants except for the NWG treatment plant which showed significant increase of nitrites after treatment. WCA and NWC indicated no significant reduction or increase of nitrates however, NWG indicated a significant increase and NWE a significant reduction. There was a significant reduction in the number of OTUs from raw water to treated water at all the treatment plants, except for the NWC at which no significant changes were observed. The total scores indicated which purification facilities were overall more effective at water purification. A higher score indicated that purification was achieved. NWE had a total score of 3, WCA and NWG had a total score of 1. NWC had a total score of -1 showing it was the least effective.

## Discussion

### Physicochemical properties of water

In the current study, we combined physicochemical properties with microbiome data to evaluate the water quality of different drinking water production plants. Treatment plants using filtration as part of the treatment process should be able to limit turbidity levels to below 0.5 NTU [16]. Turbidity in water can affect the disinfection with chlorine-based chemicals as microorganisms and pathogens can be shielded from such disinfectants if the turbidity exceeds this limit [9, 10]. In the present study, turbidity at NWE and WCA were within the limit of 0.5 NTU. NWC and NWG had turbidity levels that were slightly above 1 NTU however, based on the South African water quality guidelines, water with such levels of turbidity is still safe to drink although there is a moderate chance of adverse aesthetic effects. There is also a moderate chance of infectious disease transmission. The NWC treatment plant showed a significant increase in turbidity from raw to treated water (Table 2, Fig 1). This phenomenon could probably be ascribed to the treatment process at this plant. According to the manager of the system, the rapid sand filtration system operates in such a way that treated water is collected in a sump where it is not left for long enough so that the suspended particles can settle. The suspended material probably accounts for the significant increase of turbidity from raw to treated water. TDS concentrations below 600 mg/L in drinking water is considered to be good [29]. WCA was the only DWTP that exceeded this recommended concentration. When TDS concentrations exceed 1000 mg/L, water becomes aesthetically compromised [29]. However, in accordance with the South African National Standards [7] TDS concentrations of 1000 mg/L in drinking water has no likely health effects, even taking into account higher water consumption during very warm climatic conditions. Thus, TDS at the WCA (664–833 mg/L) was in line with the South African National Standards and falls into the category of fair TDS concentrations based on WHO guidelines.

Nutrients (nitrates and phosphates) were detected in the water after treatment and in the distribution systems. These compounds are associated with microbial growth in treated water as well as in the distribution systems [30], and they may favour biofilm formation [31]. The NWC treatment plant showed a slightly higher phosphorus in treated water compared to raw water. Regardless of the noted increase, drinking water from all the treatment plants did not exceed the WHO recommended maximum level of 5 mg/L. Nitrogen compounds such as nitrates and nitrites are interchangeable components within water environments [32]. Their levels are often associated with anthropogenic activities and their origins could be from agricultural runoff (fertilizers, pesticides) and urbanization (effluents from municipal and industrial wastewaters [33]. The source water for all the plants, was likely to be impacted by such anthropogenic activities (Table 1). Overall, nitrate concentrations were low at all the DWTPs. In a study done by Almdar et al., 2009, nitrate concentrations in the drinking water were low (2.40 mg/L– 2.80 mg/L) while the nitrite concentrations were high [32]. Similar results were observed in the current study. Nitrates and nitrites play an essential role in the maintenance and development of microbial communities [34]. pH and temperature are intrinsically linked to the physicochemical and biological reactions in water. A rise in temperature would generally increase the chemical reactions, metabolic- and growth rates of microorganisms which can also increase the turbidity. However, it does not have direct adverse effects on human health [35]. The normal range of pH for surface waters is 6.5–8.5 [36], which is also the Environmental Protection Agency (EPA) recommended pH range that municipality water suppliers must keep. In this study, the treated water was within these guidelines.

Low free chlorine concentrations were observed in the distributed water of all the DWTPs. This was similar to a number of studies which also suggested that low free chlorine concentrations can cause pathogens to survive through the distribution system [37, 38]. Thus, it is important to control and monitor free chlorine concentrations regularly within DWTPs. The physicochemical conditions (nutrients, suspended solids, pH, and temperatures) were such that an active microbial population could be sustained. Variations in the levels of these and other parameters associated with anthropogenic activities could impact the community composition of the aquatic systems.

### 16S rRNA gene profiling

Taxonomic profile analysis indicated that treatment of source water significantly influences the microbial structure of treated water. This was mainly indicated by a great decrease in the number of bacterial phyla in treated water in comparison to raw water (Fig 2). During drinking water treatment, a number of disinfectants such as chlorine, monochloramine and ozone are used to eliminate pathogenic microorganisms. Although these treatments are largely effective, some microbes can survive and proliferate in the drinking water system. A number of studies have indicated the presence of diverse microbes in drinking water distribution systems [39–41]. In our study, all treatment plants used chlorination during the disinfection step; in addition, the NWG treatment plant applies both chlorination and ozone (Table 1). In this case the ozonation is part of the treatment options, particularly to oxidize manganese [42] and not as a disinfection step. Fig 2 indicates that some of the microbes survived the treatment process and could be found in the treated water and the distribution system which is consistent with the previous studies [39, 41]. In the present study treated water was also dominated by *Proteobacteria* and *Planctomycetes*. At the NWG and NWE treatment plants, end user water was also sampled and *Proteobacteria* and *Firmicutes* dominated at these plants, respectively.

Drinking water sources play an important role in the overall composition of final drinking water [43]. This was demonstrated for the WCA and NWC treatment plants were both PCoA and dendrogram showed that the microbial community in treated water was more similar to the source water (Fig 4). NWE and NWG raw water clustered together showing similarities in the microbial communities of their source water, which was also supported by no significant differences between the phyla from these treatment plants (Fig 3). Their treated water also clustered together in agreement with the fact that source water shapes the microbial community of the treated water regardless of the treatment process. Variation of bacterial communities in source water had been shown to be a function of land use and water quality [44]. This was true for all the treatment plants, particularly NWE and NWG that clustered together (Fig 3). Similar anthropogenic activities; urbanisation, mining, agriculture and informal sectors (Table 1) are likely to impact the source waters of the two treatment plants. Moreover, the physicochemical properties of their raw water were not very distinct (Tables 2 and 3). Though WCA and NWC are likely to be impacted by agriculture, the physicochemical parameters of their raw water were significantly different accounting for the variation in the microbial communities (Tables 1–3).

From the OTUs in treated and distributed water, we detected signatures of potentially pathogenic bacteria which included *Acinetobacter*, *Clostridium*, *Legionella*, *Serratia*, *Pseudomonas* and *Tatlockia*. Some of the signatures identified up to species level are shown in S1 Table, *L*. *pneumophila* was the only species which is included in the US EPA bacteria of concern in water [45] and is the leading cause of pneumonia worldwide [46]. However, only the NWC treated water had *L*. *pneumophila* OTUs (four in total). This value might be very low for causing any illness as risk associated with ingestion of about 6.9x101–3.8x10^2^ per single event of 1 litre consumption may lead to 1 in 10,000 risk [47]. The genus *Clostridium* includes several significant pathogens. In Finland, gastroenteritis outbreak resulting from distributed water contaminated with *Clostridium difficile was* reported by [48]. Genus *Serratia* was present at NWC and NWG treated water, each having one OTU identified as *S*. spp. In addition NWG had ten OTUs classified as *S*. *marcescens* which is a well-known opportunistic pathogen. *S*. *marcescens* had been associated with urinary tract infections and catheter-associated bacteraemia [49]. *Pseudomonas* spp. and the other potential pathogens were reported in waterborne outbreaks in the United States between 2007 and 2008 [50]. However our results should be interpreted with caution as pathogens are known to harbour strain specific virulence factors, thus quantification of pathogenic taxa based on the occurrence of a biomarker such as 16 rRNA may not correlate to public health risk [51].

A number of sequences retrieved from the predicted metagenomes were associated with bacterial groups or genes that are of concern when it comes to public health. The NWC treatment plant had significantly (P < 0.05) higher proportion of predicted pathways associated with shigellosis, pathogenic *Escherichia coli* and *Vibrio cholerae* infection (S2 Fig). These were also present at all the other treatment plants particularly in raw water. Although treated water had a significantly lower proportion of these functional categories, their presence in source water should serve as a warning of the potential hazards. A study by Probert et al., 2017, have provided evidence of contaminated stream water as a source of *Escherichia coli* O157 related illness in children [52]. Predicted metagenome analyses also predicted the presence of beta-lactam resistance at all the treatment plants, as well as all water compartments (S2 Fig). The World Health Organization (WHO), has listed antibiotic resistance as a great threat to human health, and recently launched an action plan on Antimicrobial Resistance (AMR). Knowledge of the spread and distribution of AMR through research is one of the main objectives of this action plan. Antibiotic-resistant bacteria makes the treatment of community acquired infections very challenging, and their presence in drinking water is a cause for concern. Studies have also detected the presence of beta-lactam resistance bacteria in drinking water among other forms of resistance [53, 54].

Cyanobacterial species produce cyanotoxins which include microcystin, anatoxin, cylindrospermopsin [55] thus their presence especially in drinking water is highly undesirable. Treated water at the NWC, NWE and NWG treatment plants had relative abundances of Cyanobacteria (Cyanobacteria-like sequences) which were 3.83, 9.83 and 28.32%, respectively (Fig 2). Due to their similarity to chloroplast rRNA gene sequences, it is difficult to correctly classify cyanobacteria using 16S rRNA sequencing [56]. However, [40, 43] also detected cyanobacteria in drinking water using 16S rRNA gene clone libraries. Thus, the presence of Cyanobacteria-like sequences in drinking water from this study also echoes the presence of cyanobacteria in the drinking water distribution system.

Urbanisation, agriculture, mining and other anthropogenic activities have been reported for contaminating source water with a number of xenobiotics [57]. In the current study, one or more of these activities were likely to impact the source water (Table 1). Predicted metagenomes using PICRUst revealed the metabolism of atrazine, DDT, polycyclic aromatic hydrocarbon degradation (S2 Fig). Taxonomic to phenotype mapping of the OTUs using METAGENassist also predicted the presence of atrazine metabolism, degradation of aromatic hydrocarbons, naphthalene degradation, methane oxidation, chlorophenol degradation (Fig 5). Atrazine has been recently linked to pre-term birth effects [58], endocrine disruption, cancer and reproductive complications [59]. The presence of atrazine metabolism in treated water from this study (Fig 5) should serve as a warning sign to the potential hazards imposed to drinking water by the agricultural activities near source water. Furthermore, functional analysis indicated that basic microbial metabolism did not vary considerably between treated and source water (Fig 6 and S1 Fig). The different treatment plants showed varying trends of the abundance of basic metabolism between treated and raw water. This suggests that treatment process might not greatly affect some of the basic cellular process essential to bacteria, though some stress related genes might be upregulated [53]. Taxonomic to phenotype mapping reveals complex metabolic pathways (Fig 5) which includes carbon fixation, chitin degradation, chlorophenol degrading and atrazine metabolism, amongst others. These pathways indicate the key biogeochemical processes in source and treated water and perhaps could serves as an indicator of *in situ* biodegradation process potential in source and treated water. The question arises whether the presence of the pathways can be exploited to accelerate pollutant clean-up [60].

A scoring system based on significant changes from raw to treated water was used to establish physicochemical parameters and microbial abundance reduction capabilities of various water purification facilities (Table 4). We propose that this approach could be used in future studies that are investigating the effectiveness of drinking water treatment plants in reducing substance in their raw water. In some previous studies such an approach was lacking. A study by [61] evaluated the removal capabilities of natural organic matter (NOM) from South African water treatment plants. Even though the authors could compare the treatment plants for their ability to remove NOM, no statistical significance was used in the comparisons. A study by [62] also compare reduction of the same substance (NOM) during water purification processes at various plants but a similar lack of statistics was evident. Water purification plants are not always efficient in removal of all dissolved water constituents. The statistical method used in this study makes it possible to establish if reductions or increases were not only significant for a specific DWTP, but can be used to compare various plants. More importantly, the system allows for combined evaluation of physicochemical parameters and microbiome data.

The application of Next Generation Sequencing (NGS) for microbial detection has a number of limitations. NGS-based methods cannot differentiate between viable and dead bacterial cells thus in disinfected water, they may have poor comparability to culture-based methods [63, 64]. To overcome such limitations, it is crucial that NGS methods are combined with culture based methods which can provide an extra dimension of the cell viability. Kishor et al., 2019 showed that combining NGS methods and conventional methods is an effective way to evaluate water quality, the different methods will complement for the limitations of the other [65]. The presence of highly conserved 16S rRNA genes in some family and genera could lead to limited taxonomic resolution [66]. To circumvent such limitations, NGS method can be complemented with species-specific methods such as qPCR. Regardless of the limitations of NGS studies, they provide insights into community microbial structure which no other methods can provide. In our current study, removing the OTUs from the scoring system (Table 4) will not change the results of the effectiveness of the water treatment plants in this study. This suggests that even if the NGS data might have false positives, they did not significantly influence results of this study.

## Conclusions

This study gives integrated insights into the microbiome and quality of the source water, treated as well as distributed water, allowing observations of microbial-mediated processes. At the same time it evaluates the efficacy of the water treatment process used, and provides warning of the potentially looming hazards. It also adds to the baseline for monitoring perturbations in source and drinking water microbiome, which will be essential for establishing effective water treatment methods in the future. However, it is important to take into consideration the possibility of dead but intact cells as well as free environmental DNA, especially after water treatment to have an impact on the microbiome results. Even so, the data demonstrate that raw water quality is intertwined with the quality of final produced water but further to this, it also impact on the microbiome of the drinking water. We devised a method which combines physicochemical properties and microbiome data to evaluate the efficacy of various water treatment plants. This method could be applied in future studies, and it will be important to also add outgroups such as highly contaminated or pure water, so as to evaluate the methods.

## Supporting information

S1 Table Detected genera known to contain pathogenic species. (TIF)

S2 Table Linking samples to their NCBI identifiers. (TIF)

S1 Fig Distribution of the PICRUst predicted core functions among the various compartments of all the DWTPs. (A) Distribution in raw water. (B) Distribution in treated water. (C) Distribution in distributed water.
(TIFF)

S2 Fig Statistical analysis of the distribution of some of the PICRUst predicted pathways. (A) Polycyclic aromatic hydrocarbons. (B) beta-Lactam resistance. (C) Shigellosis. (D) *Vibrio cholerae* infection.
(TIFF)
