# Supplementary figures and images for: Combining physicochemical properties and microbiome data to evaluate the water quality of South African drinking water production plants
Source: PLoS One. 2020 Aug 13;15(8):e0237335. doi: 10.1371/journal.pone.0237335 (PMC7425920; doi:10.1371/journal.pone.0237335)

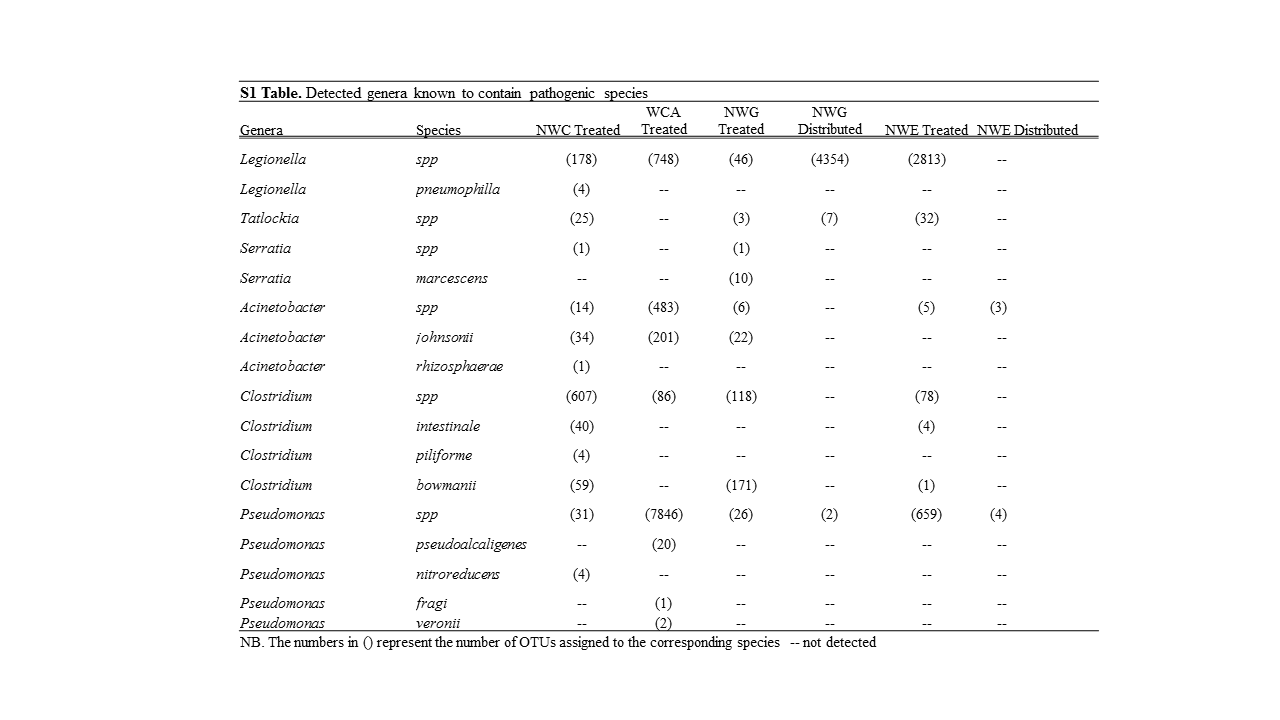

Supplement: S1 Table — (TIF) [file pone.0237335.s001.tif]

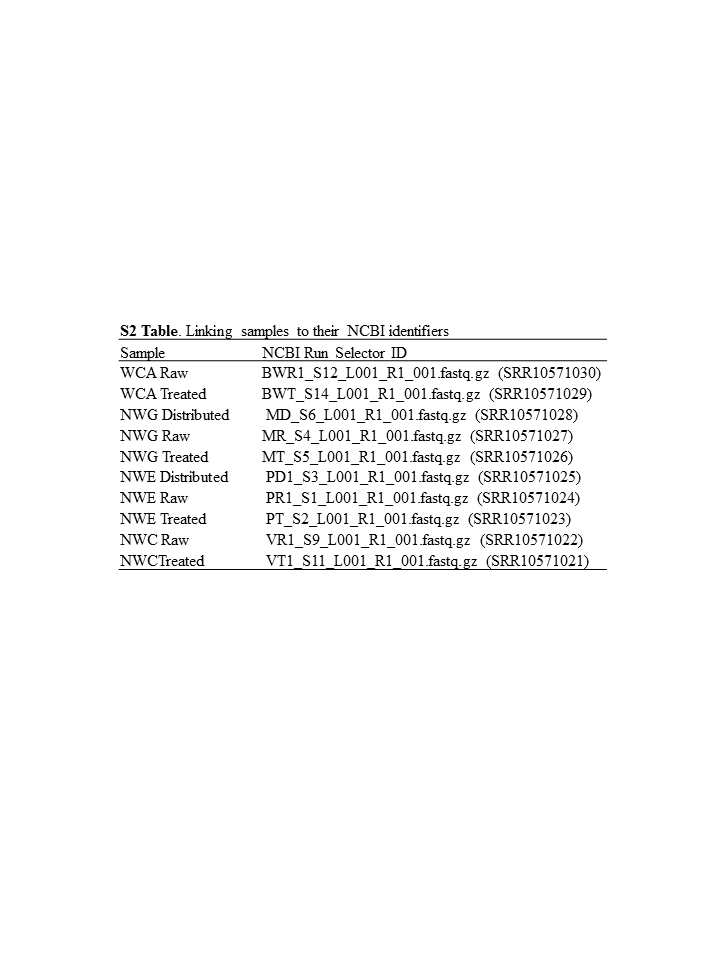

Supplement: S2 Table — (TIF) [file pone.0237335.s002.tif]

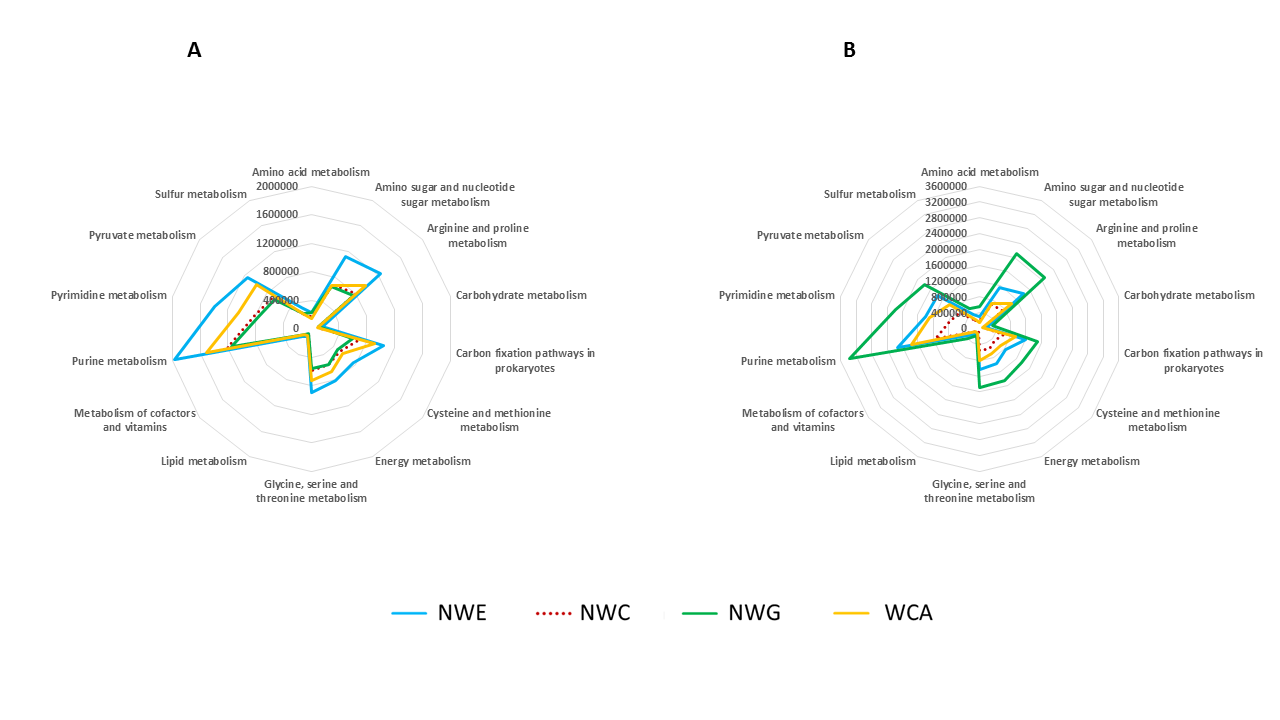

Supplement: S1 Fig — (A) Distribution in raw water. (B) Distribution in treated water. (C) Distribution in distributed water. (TIFF) [file pone.0237335.s003.tiff]

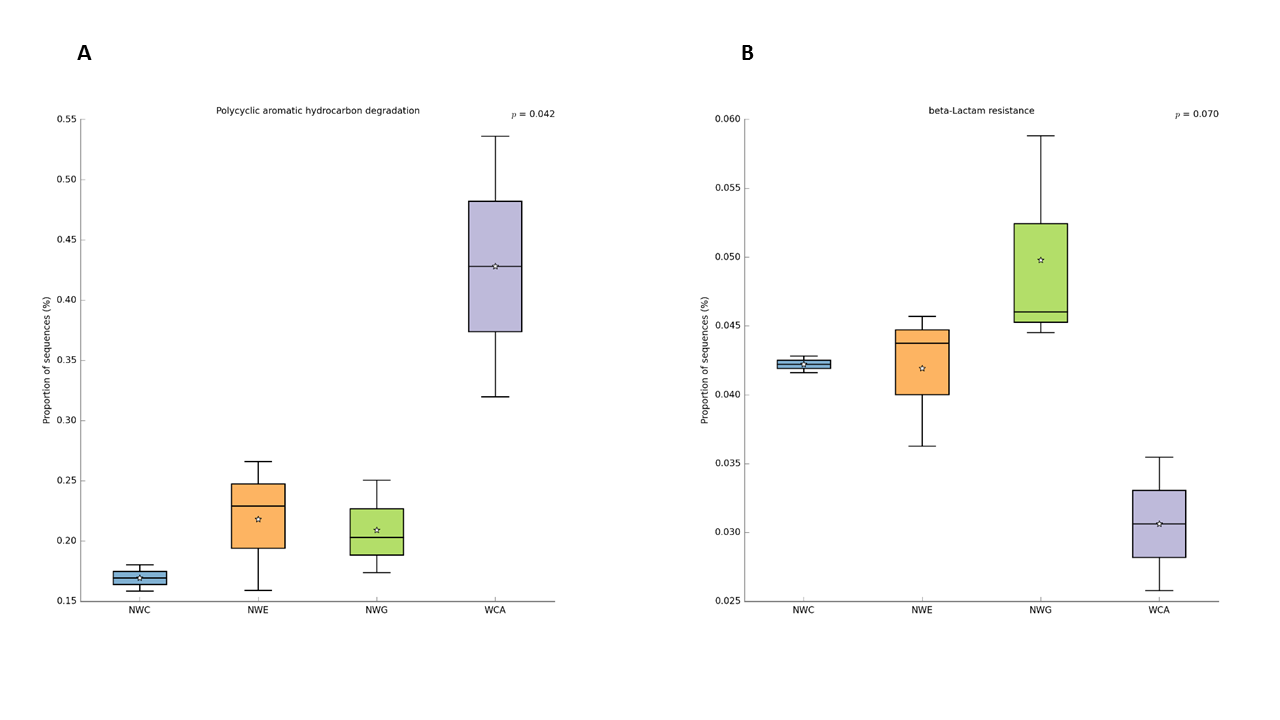

Supplement: S2 Fig — (A) Polycyclic aromatic hydrocarbons. (B) beta-Lactam resistance. (C) Shigellosis. (D) Vibrio cholerae infection. (TIFF) [file pone.0237335.s004.tiff]
